# Supplementary material for: Ethical considerations related to drone use for environment and health research: A scoping review protocol
Source: PLoS One. 2024 Jan 31;19(1):e0287270. doi: 10.1371/journal.pone.0287270 (PMC10829986; doi:10.1371/journal.pone.0287270)
Supplement: S1 File — (DOCX) [file pone.0287270.s001.docx]

**Supplementary**

**Definition "drone(s)"**: Aircraft without a pilot or passengers on-board that can be controlled remotely. Generally, not operating higher than 120m in altitude [(18)](https://www.zotero.org/google-docs/?IdaADo). Many different drone models are available on the market, and a rough distinction in functionality can be made between fixed-wing and rotor drones (**Figure 2**) [(19,20)](https://www.zotero.org/google-docs/?g1Wlun). The term "drone" is most used by the general public, whereas "unmanned aerial vehicle/system (UAV/UAS)" is preferred by aviation professionals. Following recommendations by Wang et al., the terms RPA/RPAS are not used as synonyms for drones, as these are more commonly used within military applications [(7)](https://www.zotero.org/google-docs/?48P783).

**Figure 2.** **Images of two main drone types.** A) Example of a rotor-drone (JRP Studio/Shutterstock.com), B) Example of a fixed-wing drone (Adzem/Shutterstock.com).


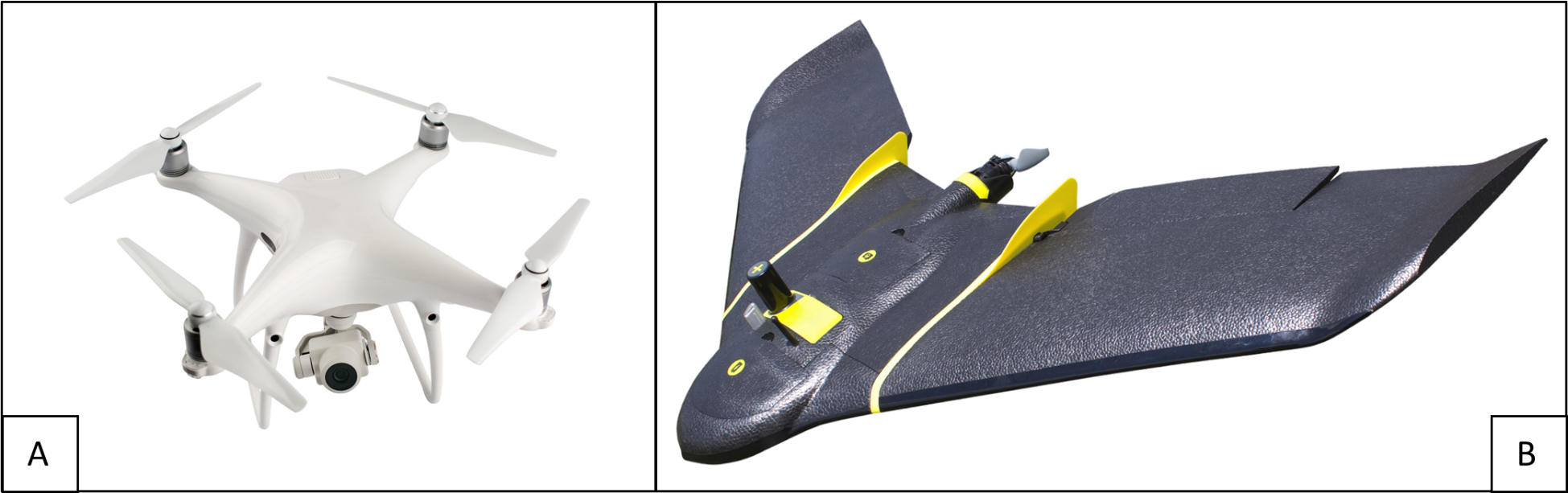


**Definition "environmental monitoring"**: Any activity that involves recording aspects of the environment without physically altering the environment. Some drone models come with a camera attached as a standard issue to take multispectral imagery. A more comprehensive overview of applications and sensors used in environmental monitoring is described by Gallacher *et al*. and within a perspective paper by Asadzadeh *et al*. [(5,19)](https://www.zotero.org/google-docs/?ptuhtd). This includes but is not limited to sensors to measure atmospheric composition, meteorology, water quality, and sound.

**Definition "ethical considerations"**: A set of moral principles guiding decision-making within environmental and health research involving interaction with human populations. Values, norms, virtues, or ethical dilemmas that are shared/expressed by the researcher or the community they are interacting with regarding the use of drones in a health or environmental research setting. Descriptions of the behaviour of researchers or community members, or interactions between researchers and community members, reflecting that they are acting on moral principles surrounding drone use in an environmental and health research setting.

**Definition “ethical decision-making”:** No specific theoretical model for ethical decision-making is assumed within the context of this scoping review protocol. It is loosely defined as the process of translating a set of heuristics rooted in the subject´s (within this protocol usually referring to a researcher) ethical values, moral principles, and experience, into actions when faced with an ethical dilemma, taking into consideration external situational factors.

**Definition “research areas”:** geographical extent of the location in which a research project is being conducted. Specifically, when pertaining to drone research; the geographical extent over which drone flights are being conducted.

**ELSI principles used by Wang *et al*.**

1. Minimising harm
2. Maximising welfare
3. Substantive justice
4. Procedural justice
5. Respect for individuals
6. Respect for communities
7. Regulatory gaps
8. Regulatory dysfunction
9. Perceptions of researchers and humanitarian organisations,
10. Relations between humanitarian organisations and industry
11. The identity of humanitarian organisations
